# Third Generation Biofuels via Direct Cellulose Fermentation

**DOI:** 10.3390/ijms9071342

**Published:** 2008-07-22

**Authors:** Carlo R. Carere, Richard Sparling, Nazim Cicek, David B. Levin

**Affiliations:** 1 Department of Biosystems Engineering, University of Manitoba, Winnipeg MB, Canada R3T 5V6; 2 Department of Microbiology, University of Manitoba, Winnipeg MB, Canada R3T 5V6

**Keywords:** biofuels, ethanol, hydrogen, cellulose, fermentation

## Abstract

Consolidated bioprocessing (CBP) is a system in which cellulase production, substrate hydrolysis, and fermentation are accomplished in a single process step by cellulolytic microorganisms. CBP offers the potential for lower biofuel production costs due to simpler feedstock processing, lower energy inputs, and higher conversion efficiencies than separate hydrolysis and fermentation processes, and is an economically attractive near-term goal for “third generation” biofuel production. In this review article, production of third generation biofuels from cellulosic feedstocks will be addressed in respect to the metabolism of cellulolytic bacteria and the development of strategies to increase biofuel yields through metabolic engineering.

## 1. Introduction

With world energy consumption predicted to increase 54 % between 2001 and 2025, considerable focus is being directed towards the development of sustainable and carbon neutral energy sources to meet future needs [1]. Biofuels are an attractive alternative to current petroleum-based fuels as they can be utilized as transportation fuels with little change to current technologies and have significant potential to improve sustainability and reduce greenhouse gas emissions. Liquid (ethanol, biodiesel) or gaseous (methane or hydrogen) biofuels are derived from organic materials such as starch, oilseeds and animal fats, or cellulose.

Cellulose is the most abundant biopolymer in the world [2]. Discarded cellulosic biomass derived from forestry, agriculture, and municipal sources are potential feedstocks for the synthesis of biofuels (and other value-added bioproducts) that could displace fossil fuel consumption and reduce greenhouse gas emissions [3, 4]. The crystalline structure of cellulose, however makes it difficult to hydrolyze. Conventional production of ethanol or H_2_ from cellulose via fermentation involves a complex process of pre-treatment including: (i) cellulase production, (ii) hydrolysis of cellulose and hemicellulose (if present), followed by (iii) fermentation of hexose sugars generated by cellulose hydrolysis and pentose sugars generated by hemicellulose hydrolysis (if present). Current strategies to produce fuel ethanol from cellulose, referred to as “second-generation” biofuels, utilize simultaneous saacharification and fermentation (SSF) or simultaneous saacharification and co-fermentation (SSCF) [5, 6]. Both SSF and SSCF require extensive pre-treatment of the cellulosic feedstock by steam-explosion and/or acid treatment, followed by addition of exogenously produced cocktails of cellulolytic enzymes to hydrolyse cellulose chains and release the glucose monomers required for fermentation.

Consolidated bioprocessing (CBP) is an alternative processing strategy in which cellulase production, substrate hydrolysis, and fermentation are accomplished in a single process step by microorganisms that express cellulolytic (and hemicellulolytic) enzymes [5–7]. CBP offers the potential for lower biofuel production costs due to simpler feedstock processing, lower energy inputs (and therefore better energy balance), and higher conversion efficiencies than SSF based processes. CBP is an economically attractive near-term goal for process for “third generation” biofuel production [5–7].

Successful development of “third generation” biofuels depends heavily on a detailed understanding of the metabolism of cellulolytic bacteria. In this paper the application of metabolic engineering strategies to improve biofuel yields (ethanol and hydrogen) will be discussed. Particular emphasis will be placed on the utilization of cellulose as an available feedstock and the metabolism of cellulolytic Clostridia.

## 2. Biofuels and Fossil Fuels

### 2.1. Environmental concerns

The extraction, processing and combustion of fossil fuels contributes to the pollution of soil, air and water and thus is hazardous to the environment and to public health [8]. In 1996, the Intergovernmental Panel on Climate Change (IPCC) reported the earth'smean surface temperature had increased 0.2 °C per decade since 1975 and asserted anthropogenic greenhouse gasses (GHG) as the primary contributor to the observed change. Greenhouse gasses include carbon dioxide (CO_2_), methane (CH_4_), nitrous oxide and various engineered chemicals including chloroflurocarbons (CFCs). These gasses prevent heat reflected from the earth'ssurface from escaping into space and therefore contribute to a global warming [9]. CO_2_ emissions contribute to the majority of the documented heat trapping capacity of the atmosphere and worldwide CO_2_ concentrations continue to increase by 0.5 % annually. The combustion of fossil fuels is by far the largest contributor to the observed CO_2_ increase and in general accounts for approximately 60 % of greenhouse warming resulting from anthropogenic sources [9]. In response to growing concerns regarding environmental degradation, biofuels have emerged as an attractive alternative to conventional petroleum-based fuels. The utilization of fuels derived from biomass can significantly reduce greenhouse gas emissions. In 1998 Brown *et al*. reported that the utilization of bioethanol as an automotive fuel could reduce CO_2_ emissions by 60–90 % relative to conventional petroleum fuels. CO_2_ released during the combustion of bioethanol is recycled through the photosynthetic process resulting in no net increase to CO_2_ levels. The combustion of neat ethanol (E100), however, contributes to the emission of aldehydes, particularly acetaldehyde, which is between 2 to 4 times lower in gasoline emissions. Alternatively, the utilization of hydrogen as a transportation fuel would significantly reduce GHG emissions. Hydrogen fuel cells directly convert chemical energy to electrical energy without the need of combustion. In theory this conversion can achieve efficiencies approaching 90 %. While a H_2_ fuel cell produces only water, it is noteworthy that the biological production of H_2_ via dark fermentation gas does involve production of large quantities of CO_2_ [10–12].

### 2.2. Bioethanol

Bioethanol is the most widely used liquid biofuel. In 2004 worldwide production of bioethanol reached 41 billions litres. The largest producers in the world are Brazil (37%), the United States (33%), and Asia (14%). Production of bioethanol from sugarcane in Brazil reached 16.4 billion liters (2004), accounting for nearly 18% of the country'sautomotive fuel needs. In Brazil, ethanol-powered and flexible-fuel vehicles are manufactured for operation with hydrated ethanol, an azeotrope of ethanol (around 93% v/v) and water (7%) [13–15]. As a result of this, coupled to the development of domestic deep-water oil sources, Brazil has achieved complete self-sufficiency in oil [9]. Recently the United States (US) surpassed Brazil as the world'slargest producer of bioethanol (18 billion litres compared to 16 billion litre, respectively).

The demand for bioethanol is expected to increase dramatically until 2010. In 1999 the US signed an executive order specifying a tripling in the production of biobased products and bioenergy by the year 2010. As a consequence, US oil imports will be reduced by nearly 4 billion barrels over that time. Efforts to decrease GHG emissions are expected to spur the production of renewable energy sources by 6% within the European Union by 2010 [16]. In France, the approval of a clean air act could increase ethanol production to 500 million litres. Similar projects in Spain, Sweden and the Netherlands are expected to increase the utilization of ethanol to account for 15 % of transportation fuels by 2010 [17].

Fuel ethanol can be used in a variety of ways. Ethanol is commonly used as an oxygenated fuel additive to reduce emissions of carbon monoxide, nitrous oxides and hydrocarbons [18]. Numerous common ethanolic motor-fuel formulations are being used with increasing frequency (Table 1) [18]. Ethanol has a higher octane rating than petroleum fuels enabling combustion engines to run at higher compression ratios and thus give superior net performance [18]. In addition, ethanol exhibits higher vapour pressure and heat of vaporization than gasoline and therefore increased power outputs are observed while using ethanol [16].

Bioethanol is produced by converting sugars directly from crops like sugarcane or sugar beets, indirectly through starch from corn, wheat, potatoes, or cassava, or through cellulose from biomass, into ethanol via fermentation followed by distillation [19]. Improving ethanol yield is critical to the establishment of a viable industry and is fundamentally tied to increasing throughput and reducing costs associated with production. During the fermentation of sugarcane, up to 10% of available sugar can be diverted away from ethanol and converted to glycerol and succinic acid. Attempts to improve ethanol yields by reducing the synthesis of these unwanted metaboites has resulted in reduced yeast competitiveness within fermentors [18]. Glycerol production is coupled to acid, ethanol, and temperature induced stress conditions. The synthesis of glycerol occurs in response to osmotic stress and therefore likely has an essential role in cell viability. Although yeasts with improved properties such as ethanol and temperature tolerances have been genetically engineered, such strains are not yet used widely by the fuel ethanol industry [16].

### 2.3. Biohydrogen

Hydrogen gas (H_2_) is a clean fuel that possesses a high energy content per unit weight (122 KJ g^−1^) and does not contribute particulate or greenhouse gas emissions into the atmosphere upon combustion [20]. Hydrogen can be produced by nuclear or fossil fuel mediated electrolysis of water, coal gasification, and steam reformation of natural gas or through the action of biological systems. Production of H_2_ using fermentative biological processes is potentially the most attractive of these strategies as it is not as energy intensive as other means and could potentially utilize refuse or agricultural waste-streams as the raw material [3, 11]. Periodic crises in the supply and price of fossil fuels has drawn attention to the fact that renewable energy sources are the only long-term solution to future energy requirements. Consequently, improving H_2_ yields, reducing production costs and developing technologies to exploit this fuel are being actively investigated. Attainable biohydrogen yields and the potential for increasing these yields in the future are not well understood. Several recent studies focusing on saccharolytic species of clostridia have begun to investigate potential H_2_ yields.

The biological production of H_2_ is fundamentally dependent on hydrogen evolving enzymes. Hydrogenases constitute a family of enzymes found throughout the bitota. These enzymes catalyze the reversible oxidation of hydrogen gas: H_2_ ↔ 2H^+^ + 2e^−^. While the host organisms of a hydrogen-producing hydrogenase, such as the CpI hydrogenase in *Clostridium pasteurianum*, will typically use it to dispose of excess electrons, considerable research has recently focused on the potential for these enzymes to produce hydrogen as an energy source. If harnessed properly, hydrogenases and/or hydrogenase-containing organisms could be used to supply affordable and renewable H_2_ to be used as an energy fuel.

Polymerase chain reaction (PCR) targeting 16S rDNA and known hydrogenase nucleotide sequences has been used to confirm the presence of clostridial species within anaerobic biohydrogen fermenting reactors [21]. Samples taken from the exponential-phase of growth and subsequent reverse-transcriptase (RT) PCR and DNA sequence analyses revealed that the H_2_ producing strains either expressed a *Clostridium pasteurianum*-like or *Clostridium saccharobutylicum*-like hydrogenase [21].

The potential for H_2_ production by *Clostridium thermolacticum* during continuous fermentation of lactose was explored using a waste-stream generated by the cheese industry. Approximately 150,000 tonnes of milk permeate are produced each year within Switzerland as a by-product of cheese production. Given its high biochemical oxygen demand for biodegradation, the effluent must be treated prior to release into the environment [22]. Hydrogen formed during lactose fermentation by *C. thermolacticum* was measured during continuous culture at different dilution rates and pH levels. Although acetate was the major metabolite formed as a result of lactose fermentation, H_2_ was evolved as a by-product. Collet *et al*. [22] found that H_2_ production was maximized at high dilution rates by maintaining a pH above 7.0. They concluded that lactose fermentation by *C. thermolacticum* represents a cheap alternative to biohydrogen production that makes use of an otherwise polluting waste-stream.

## 3. Cellulose Feedstocks and Cellulolytic Bacteria

Biofuels may be produced from the sugars of a variety of different feedstocks, including food crops and cellulosic substrates. Food crops such as sugarcane and sugar beets contribute to 60% of the world'sbioethanol production and contain large amounts of sucrose [16]. Other crops, including corn and cereal crops like wheat, contain starch that is then converted into glucose during fermentation. The expansion of biofuels production, particularly in the United States, together with increased world-wide demand for grains and increased energy costs, has led to drastically higher grain prices.

The total worldwide harvest of grains in 2006 was estimated at 1.79 billion tonnes, 4% short of the estimated consumption of 1.85 billion tonnes [23]. This is the sixth time in the last seven years that world grain production has been lower than world consumption. As a result, world carryover stocks of grain have fallen to only 57 days of consumption, the lowest level since the mid-1970s [23]. In the US, competition for land and other resources used to produce corn for fuel ethanol already has led to restrictions in land use for oilseed production and consequently higher prices associated with lower production levels. The pursuit of lower biofuels production costs and competition with traditional food crops has led many to consider cellulosic substrates as a potential feedstock.

### 3.1. Cellulose

Cellulose is the most abundant bio-polymer on earth; an estimated 7.5×10^10^ tons are annually synthesized through photosynthetic processes [2]. Found primarily in plant cell walls, cellulose is embedded in a hetero-matrix composed of xylan, other hemi-celluloses and lignin. Specifically, cellulose is a linear, insoluble biopolymer composed of repeating β–D-glucopyranose residues linked by β-1,4 glycosidic bonds. In contrast to other glucan polymers, such as starch, the repeating unit of cellulose is not glucose, but cellobiose, a disaccharide. Cellulose exhibits a high degree of polymerization: the individual glucan chains, or cellodextrins, can reach lengths of greater than 25,000 glucose residues [24].

Cellulose produced by plants is composed of both highly amorphous regions containing large voids and other irregularities as well as tightly packed crystalline regions. Cellulose, because it is resistant to most forms of degradation, accumulates within the environment. It has been estimated that approximately half of the carbon fixed annually within terrestrial ecosystems is stored as cellulose. Cellulose synthesis is primarily associated with plants, however some animals, bacteria and algal species can also produce the polymer [25]. Organisms that are capable of degrading the polymer and utilizing it as a source of carbon are ecologically very important. Cellulose is generally degraded into H_2_O and CO_2_ in aerobic systems while in anaerobic systems CH_4_ and H_2_ are also produced. Although, most cellulose is degraded in aerobic environments, 5 to 10% is degraded under anaerobic conditions by a range of physiologically diverse bacteria. Among these cellulolytic bacteria class Clostridia have been best studied and characterized. These bacteria are ubiquitous in anaerobic soil environments, form endospores and digest cellulose via an exocellular enzymatic complex called a cellulosome, converting cellulose into several different metabolites.

Cellulolytic species are found within the phyla Thermotogae, Proteobacteria, Actinobacteria, Spirochaetes, Firmicutes, Fibrobacteres and Bacteroids. Of these, approximately 80% of the isolated cellulolytic bacteria are found within phyla Firmicutes and Actinobacteria [26]. The majority of the gram-positive cellulolytic bacteria are found within Firmicutes and belong to the class Clostridia and the genus *Clostridium*.

### 3.2. Detection and enumeration of cellulolytic bacteria

As the primary and rate-limiting step involved in waste degradation, cellulose breakdown is an important step in the development of strategies to treat municipal solid waste [27]. O'Sullivan *et al*. [28] investigated enriched cellulolytic microbial communities within anaerobic batch reactors in order to determine the dynamics of cellulolytic bacterial populations during the fermentation process. In fermentation experiments conducted under mesophilic temperatures, 80 % of the cellulose was solubilised within 20 days [28]. FISH analysis revealed the bacteria grew as surface-attached biofilms. Fluctuations in relative abundance of the three clostridial groups did not have a significant impact on the rate of cellulose degradation. Over the initial incubation period, the sum of the three target groups of bacteria accounted for more than 99% of the total bacteria present. After cellulose depletion, however, the proportion of the target bacteria was reduced to 13%. In general, *C. thermocellum*-like bacteria were always the most abundant group throughout the study, while *C. sterocorarium* and *B. cellulosolvens*-like bacteria also accounted for a significant proportion of the community. The lack of evidence linking clostridial population dynamics to the rate of cellulose degradation suggests that cellulose solubilisation rates are dependent on the amount of colonized surface area and biofilm architecture.

Within cellulose rich environments, non-cellulolytic bacteria can affect the metabolic flux of cellulose hydrolyzing microorganisms. In 1977, the fermentation of cellulose and cellobiose by *Clostridium thermocellum* was investigated in the presence and absence of *Methanobacterium thermoautotrophicum* [29]. It was hypothesized that methanogenic bacteria are able to act as electron sinks by making it energetically favourable for cellulose fermenting bacteria to dispose of H_2_ instead of other reduced products such as ethanol [29]. Known as interspecies hydrogen transfer, H_2_ produced by cellulose fermenting bacteria is oxidized to methane by methanogenic microorganisms. Co-cultures of *C. thermocellum* and *M. thermoautotrophicum* produced more H_2_ and acetic acid and less ethanol than within *C. thermocellum* monocultures. Furthermore, when grown on cellulose, the co-culture exhibited a shorter lag before growth, and cellulase activity appeared earlier within the co-culture than in the monoculture. The conversion of H_2_ to methane was complete within the co-culture and the majority of the methane produced was derived from the reduction of CO_2_ rather than acetate conversion [29]. Within the cellobiose medium, the methanogen caused only very small changes to the fermentation balance of *C. thermocellum*. The absence of free H_2_ within the co-culture grown on cellulose indicated that methanogenesis was limited by the rate of H_2_ evolution by *C. thermocellum*. The increased growth rate of *C. thermocellum* in the cellobiose culture was sufficient to result in an accumulation of H_2_. In this instance, methanogenesis within the cellobiose co-culture was limited by the growth rate of *M. thermoautotrophicum* [29].

Weimer & Zeikus [29] concluded that the altered fermentation patterns observed within *C. thermocellum* grown in co-culture with *M. thermoautotrophicum* was in general agreement with the concept of interspecies H_2_ transfer. The absence of significant metabolic interactions between the clostridia and the methanogen when grown on cellobiose can be explained in terms of ecological significance. In nature, the decomposition of organic matter is limited by the rate of the degradation of insoluble biopolymers such as cellulose. In this regard, soluble intermediates of anaerobic digestion, including glucose, cellobiose, and acetate are normally found in low concentrations and generally have a smaller environmental impact than readily available insoluble substrates such as cellulose. From a kinetic point of view, interspecies H_2_ transfer that influences the rate at which insoluble biopolymers are degraded may be of greater environmental consequence than transfers involving mixed-cultures grown on soluble substrates such as cellobiose [29].

## 4. The Cellulosome

### 4.1. Cellulolytic enzymes

Through the secretion of cellulases as single enzymes, as single polypeptides with multiple cellulosic domains or as extracellular multienzyme complexes, microorganisms have developed several strategies to digest cellulose. The cellulosome, which was first described within *C. thermocellum* by Lamed *et al*. [30], is a multi-component cellulolytic exocellular complex of proteins that mediates cellulose binding and degradation. Similar to other cellulose degrading strategies, the cellulosome hydrolyzes the biopolymer to its building block, the disaccharide cellobiose and cellodextrins. The cellulosome has emerged as a specialized structure that plays a significant physiological role. On the cell surface they appear as polycellulosomal aggregates promoting the adherence of the bacterium to the cellulose fibre [31]. Cellulosomes are able to hydrolyze both amorphous, and highly ordered crystalline cellulose. The degradation of cellulose is accomplished through the action of enzymes that include endo-1,4-β-glucanases and exo-1,4-β-glucanases (cellobiohydrolases).

**Figure 1.**
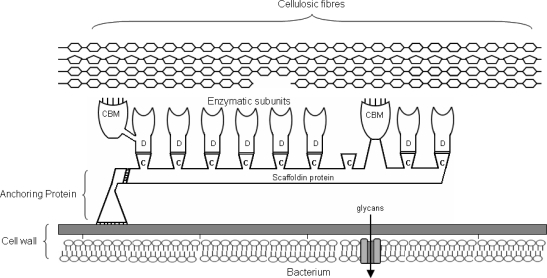
Diagrammatic representation of the cellulosome of *Clostridium thermocellum* ATCC 27405. The scaffoldin protein (CipA) is linked to the cell wall via an anchoring protein. Cohesion domains (C) located on the scaffoldin protein mediate attachment to dockerin domains (D) of various enzymatic components. Binding to cellulose is accomplished via cellulose binding motifs (CBM) associated with both the scaffoldin protein and some enzymatic components (adapted from [32]).

Endoglucanases are able to hydrolyze amorphous cellulose, carboxymethylcelluose, and phosphoric acid-swollen cellulose, producing soluble oligosaccharides that are subsequently degraded into cellobiose and glucose through the action of β-glucosidase. Cellobiohydrolases degrade cellulose by cleaving cellobiose units from the non-reducing end of a cellulose fibre [31]. A major component of the cellulosome is a large non-enzymatic polypeptide that binds and supports the enzymatic subunits of the cellulosome to the cell. This protein, referred to as scaffoldin or CipA, has no catalytic activity and is modularly organized with numerous distinct cohesion domains and cellulose binding motifs. Some cohesion domains of the scaffoldin protein permit specific binding to the dockerin domains of the various enzymatic components of the cellulosome. Other cohesion domains mediate cell attachment via cell-surface attached dockerin domains. Cellulose attachment can be mediated by cellulose binding motifs present on the scaffoldin protein or from motifs present on the various enzymatic components (Figure. 1).

### 4.2. Mechanisms of cellulose degradation

A number of studies have been dedicated to enhancing our understanding of the cellulosome. The specific and direct attachment to the substrate permits efficient competition with other non-cellulosome utilizing cellulose degrading bacteria. Close proximity of the cellulose to the cell aids in minimizing the diffusion of soluble cello-oligosaccharides into the extracellular environment and insures an efficient uptake into the cell. From an enzymatic point of view, the cellulosome promotes concerted activity and synergism of the enzymatic components. The action of endo-1,4-β-glucanases produce novel non-reducing ends for enzymatic attack by cellobiohydrolases yielding cellobiose residues. The subsequent hydrolytic action of β-glucosidase or phosphorolytic activity of cellobiose phosphorylase then converts cellobiose into glucose exclusively or both glucose and glucose-1-P residues respectively. In comparison to the hydrolysis of cellobiose, cleavage via the phosphorolytic route exhibits an apparent lower Km and leads to pathways yielding more net ATP production [7]. Although first described within *Clostridium thermocellum*, numerous other clostridial species have been shown to utilize a cellulosome as their cellulose degrading strategy. These bacteria are ubiquitous in anaerobic soil environments and include *C. cellulolyticum*, *C. acetobutylicum*, *C. cellobioparum* and C. papyrosolvens [24].

Ultra-structural observations using electron microscopy, Fourier-transform IR spectroscopy and X-ray diffraction analysis have reaffirmed that the synergistic action of the various enzymes of the cellulosome provides an advantage over cellulose degrading strategies that employ free cellulase systems [25]. In aerobic fungi, various cellulolytic enzymes are excreted as separate entities, each displaying a specific enzymatic function. In contrast, the cellulosome consists of discrete multi-functional enzymatic subunits physically attached to a central scaffoldin protein. Studies have shown that the cellulases of *C. thermocellum* are at least 50 times more active in cotton degradation than the extracellular cellulase system of the fungus *T. reesei* [33, 34].

It has been hypothesized that the rate-determining step for cellulose degradation is the initial attack. Once a cellulose fibre has undergone initial enzymatic attack, degradation occurs in a processive manner until the fibre has been completely degraded. This explains why some intact fibres are still observed after near complete solubilisation of available cellulose, it is possible that some fibres represent a sub-population that are highly resistant to the action of cellulases.

### 4.3. Regulation of cellulase synthesis

Regulation of cellulase synthesis is an important feature for the physiology of cellulolytic clostridia. The synthesis of a single cellulosome requires a substantial initial investment of ATP. The cellulosome of *C. thermocellum* is composed of more than 20 different catalytic subunits in addition to the scaffoldin protein. The regulation of expression for these proteins plays a critical role in determining cellulose hydrolysis and cell growth rates. It has been shown that the degradation of crystalline cellulose is significantly decreased within cultures that are grown concomitantly on cellobiose [35]. This suggests the expression of cellulase proteins is inhibited through a carbon catabolite repression (CCR) mechanism in which the presence of cellobiose negates the requirement for continued assembly of cellulosomes. Recently, an enzyme-linked immunosorbant assay (ELISA) based protocol was developed using an antibody raised against a common cohesion domain of the scaffoldin protein. In this study, cellulase production was inferred based on the quantity of scaffoldin observed [36]. Cellulase expression was measured in cultures grown on Avicel and cellobiose within both batch and continuous culture conditions. ELISA results revealed that a nine-fold greater quantity of cellulase was detected within cultures grown on Avicel compared to cellobiose grown cells. The inverse correlation between cellobiose concentrations and cellulase adds support to the theory that the assembly of cellulosomes is mediated by a carbon catabolite repression mechanism [37].

## 5. Metabolism of Cellulolytic Clostridia

### 5.1. Sugar uptake

The metabolism of cellulolytic clostridia has best been studied within *Clostridium cellulolyticum. C. cellulolyticum* is a mesophilic, cellulolytic bacterium that was originally isolated from decaying grass. As with most truly cellulolytic clostridia, *C. cellulolyticum* degrades cellulose via a cellulosome and releases soluble cello-oligosaccharides, principally cellobiose. The first studies of metabolic activity focused mainly on *C. cellulolyticum* behaviour such as colonization. These studies showed that following colonization of a cellulose fibre, the catalytic components of the cellulosome began the depolymerisation process and consequently soluble sugars, such as glucose and cellodextrins (*i.e.* from cellobiose to celloheptose) were released from the cellulose fibre [38]. This early work indicated that the released cello-oligosaccharides remain in close proximity to the cell between the cellulosome and the cell wall before uptake. The uptake of these sugars is a highly efficient process that utilizes an ATP-Binding Cassette transport system [39]. Uptake into the cell of the soluble sugars often occurs within seconds after their initial release from the cellulose fibre [39]. Following uptake, sugars are processed within the cytosol by β-glucosidase or cellobiose phosphorylase and cellodextrin phosphorylase proteins, producing glucose-6-phosphate residues ready for entry into the glycolytic pathway.

### 5.2. Cellulose Fermentation and metabolic fluxes in C. cellulolyticum

**Figure 2.**
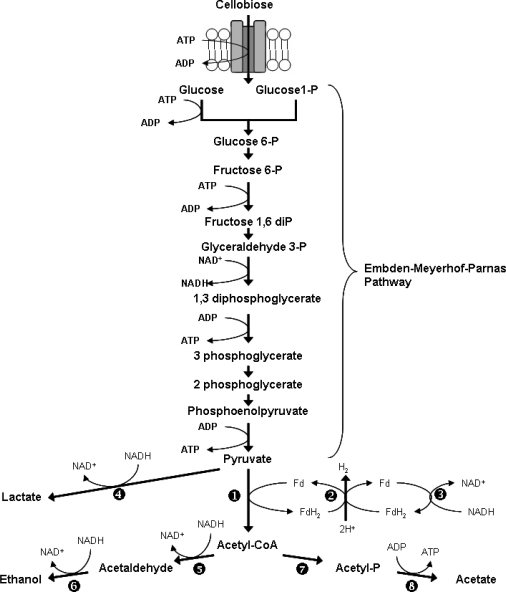
The catabolic pathway of cellobiose in *C. cellulolyticum*. 1: pyruvate-ferredoxin oxidoreductase; 2: hydrogenase; 3: NADH-ferredoxin oxidoreductase; 4: lactate dehydrogenase; 5: acetaldehyde dehydrogenase; 6: alcohol dehydrogenase; 7: phosphotransacetylase; 8: acetate kinase. [24]

As within any cell, carbon and electron flow within *C. cellulolyticum* are closely linked. Clostridia generate their ATP through the phosphorylation of the carbon substrate and the final electron acceptors are organic molecules. During glycolysis NADH is generated during the conversion of glyceraldehyde-3-phosphate to 1,3-diphosphoglycerate and afterwards a second oxidation occurs when pyruvate is converted to acetyl-CoA by pyruvate: ferredoxin oxidoreductase (POR). This reaction leads to the formation of reduced ferredoxin after which the electrons are transferred to a Fd-dependent hydrogenase and H_2_ is released [24]. NADH generated during the Embden-Meyerhof-Parnas pathway can be oxidized by NADH:Fd reductase to generate fresh reducing equivalents for the catabolic process. In conditions of high carbon flux, pyruvate overflow necessitates the oxidation of reducing equivalents through the generation of less-reduced metabolites such as ethanol and lactate (Figure 2; [40]).

Guedon *et al*. [41] demonstrated that metabolite yields in *C. cellulolyticum* depend strongly on the initial cellulose concentration and that early growth arrest was linked to pyruvate overflow. In this study, carbon flow in *C. cellulolyticum* was investigated within batch and continuous cultures using synthetic media with cellobiose as the sole carbon source. By measuring the specific rates of NAD(P)H production and consumption, the specific rates of H_2_ production, the oxidation/reduction ratio and the main products of cellobiose fermentation, Guedon *et al*. [41] were able to determine that cellobiose catabolism exceeded the rate of pyruvate consumption. This suggested that pyruvate consumption via POR was a limiting step in the glycolytic pathway. Furthermore, pyruvate overflow was tightly coupled to an observed inhibition of growth.

Previous work had suggested that growth of *C. cellulolyticum* was limited by an imbalance in the specific rates of NADH production and consumption. Intracellular NADH/NAD+ ratios as high as 57:1 had been observed within chemostat grown cultures and it was concluded that the reduction of NAD+ during ethanol, lactate, and H_2_ synthesis was limiting [42]. Although the lack of available reducing equivalents affected the pronounced growth inhibition observed, the critical bottleneck that occurred during glucose catabolism in *C. cellulolyticum* was the conversion of pyruvate to acetyl-CoA via POR. Guedon *et al*. [41] contend that *C. cellulolyticum* is not adapted to utilize carbon sources or other nutrients in excess. In natural ecosystems, habitats rarely have all nutrients in saturating quantities. Therefore, populations of cellulolytic clostridia are unlikely to ever face such a bottleneck in nature.

In contrast, a chemically defined medium contains all required nutrients for growth and therefore growth inhibitions as a result of bottlenecks in the glycolytic pathway are more likely to occur. In spite of the fact that many bacteria have developed the ability, spontaneously or through adaptation, to adjust to growth on carbon-rich media, this behaviour is not generally observed in cellulolytic bacteria [43]. Rather than adapt to habitats of high carbon availability, it is likely that *C. cellulolyticum* has evolved to optimize it'scatabolic pathways to exploit pools of low available carbon sources [41].

Continuous culturing of *C. cellulolyticum* on synthetic medium containing cellobiose as the sole carbon source demonstrated that the flow of electrons from the glycolytic pathway was balanced by the production of lactate, ethanol, and H_2_ gas. At low levels of carbon flow, pyruvate was oxidized preferentially to acetate and lactate, maximizing ATP formation. Conversely, high levels of carbon flux led to pyruvate overflow and to increased levels of ethanol and lactate production. Pyruvate overflow represents an inability of POR to support the carbon flow arriving at pyruvate from the catabolism of cellobiose. Under these conditions, electron flow from glycolysis was balanced primarily by ethanol and lactate production [41]. For the reason that lactate production was only observed within conditions of pyruvate overflow, it can be inferred that there is no competition between the lactate producing and the acetyl-CoA producing pathways.

This preference for pathways generating ATP over pathways designed to generate reducing equivalents further supports the theory that *C. cellulolyticum* rarely finds nutrient substances in high concentrations in nature. Although the optimization of cellobiose catabolism under nutrient poor conditions has been proposed for *C. cellulolyticum*, other cellulolytic species exhibit considerably different fermentation profiles. In relation to biofuels production, the metabolism of cellulolytic bacteria is highly dynamic and unique to each species. End-product yields are dependant on numerous factors (pH, carbon accessibility, redox balance, gas pressure, product concentration) and strategies aiming to metabolically engineer novel strains of bacteria for increased biofuels production should be based on a detailed understanding of the genetic, enzymatic and thermodynamic mechanisms that direct carbon flow.

### 5.3. Cellulose fermentation and metabolic fluxes in C. thermocellum

C. thermocellum is a thermophilic (optimum growth at 60 oC) bacterium that utilizes cellulose as a sole carbon source and carries out mixed product fermentation, synthesizing various amounts of lactate, formate acetate, ethanol, H_2_, and CO_2_, under different growth conditions [7]. As previously described, *C. thermocellum* expresses a cellulosome on its surface, and displays the highest known rate of cellulose degradation [6, 44, 45]. Fermentation of dilute-acid pre-treated hardwood or avicel (crystalline cellulose) in batch and continuous cultures of *C. thermocellum* were reported by Lynd *et al*. [44]. In batch cultures, ethanol and acetate were the main products, with final molar ratios of ∼2.3:1 ethanol:acetate. Lactate synthesis was also observed at low levels as the cells entered stationary phase. In continuous cultures of *C. thermocellum*, ethanol:acetate (mol/mol) ratios were ∼ 1.3:1, and substrate conversion ranged from 0.86 at a dilution rate (*D*) of 0.05/h to 0.48 at a *D* of 0.167/h for avicel and 0.75 at a *D* of 0.05/h to 0.43 a *D* of 0.11/h for pretreated wood [44].

Hydrogen and soluble end-product synthesis patterns by *C. thermocellum* in batch cultures were also investigated by Levin *et al*. [4], in batch cultures, using either cellobiose or cellulosic substrates (α-cellulose, shredded filter paper, and delignified wood fibers) at low (0.1 g/L), medium (1.1 g/L), and high (4.5 g/L) initial substrate concentrations. Cellulosic substrates produced higher total amounts of H_2_ in high substrate concentration cultures, but better H_2_ yields were observed at both low and medium substrate concentrations. Delignified wood fiber was the most effective substrate, providing an average yield of 1.6 mol H_2_ mol/glucose equvalent. Ethanol, acetate and formate were produced during exponential growth, while lactate synthesis coincided with a decrease in pH to ∼ 6.3 [4, 46, 47]. Batch cultures in which the initial pH was set at 6.3 (with cellobiose as the sole carbon source) produced only lactate and displayed very slow growth [48]. On average, the ratio of ethanol:acetate was lower (1:1.3) than that reported by Lynd *et al* [44], and stayed roughly constant under all growth conditions tested [4]. H_2_ production and yields were similar or greater in cultures containing cellulosic substrates compared with cellobiose, and H_2_ production increased with the concentration of cellulosic substrate. Thus, it appears that *C. thermocellum* metabolism is not limited by carbon flow (unlike *C. cellulolyticum*, where metabolic flux is greatly influenced by carbon flow into the cell).

Shifts in metabolic end-product synthesis patterns can be induced by the addition of acetone, sodium azide or by increasing the hydrostatic pressure of the bioreactor by adding exogenous H_2_ [49] or N_2_ gas [50]. End-product mediated metabolic shifts were also observed in *C. thermocellum* [48]. Addition of ethanol to the growth medium at the initiation of the fermentation process resulted in significant increases in H_2_ and acetate (54% and 25%, respectively). Formate addition increased H_2_ and ethanol by ∼10% each and decreased acetate production. Addition of H_2_ decreased CO_2_ and increased formate production, whereas acetate increased ethanol and decreased formate production. The addition of 10 % carbon monoxide (CO) in the bioreactor headspace led to decreased synthesis of H_2_, CO_2_, and acetate, but significantly increased in ethanol synthesis.

## 6. Metabolic Engineering

The application of recombinant DNA techniques to direct metabolism towards the production of industrially valuable substrates is an emerging field of study. Metabolic engineering seeks to “improve” cellular function through the modulation of enzymatic, transport, or other regulatory functions of the cell. In contrast to traditional strain improvement approaches that involve mutagenesis followed by the screening of colonies for a desired phenotype, metabolic engineering often involves the introduction of heterologous genes or regulatory elements that are employed to confer novel metabolic conFigureurations [51]. The introduction of genes encoding heterologous proteins, however, does not guarantee attainment of the desired phenotype. The expressed, heterologous protein must avoid proteolysis, fold and assemble correctly, and obtain appropriate prosthetic groups and/or post-translational modifications [51]. In addition to these potential barriers, successful metabolic engineering requires a detailed understanding of the factors influencing anabolic and catabolic flux (pH, redox potential, gas partial pressure, end-product inhibition) and a strategy to best exploit cellular function.

The cloning and expression of heterologous genes can serve several purposes including: 1) extending existing pathway to produce novel products, 2) engineering arrays of enzymatic activities that synthesize novel structures, 3) shifting metabolic flux towards synthesis of desired end-products and 4) accelerating a rate determining step [51].

### 6.1. Completion of partial pathways for novel product synthesis

The spectrum of substrate utilization and product synthesis is a reflection of the genetic and metabolic diversity that exists in nature. Many bacterial species, however, are inefficient from an applied perspective as oftentimes native pathways leading to the synthesis of valuable substrate are incomplete as a result of transposition, horizontal gene transfer or mutation events. One established strategy pertaining to metabolic engineering for industrially valuable substrate production involves the completion of pre-existing pathways. As an example, the final precursor to ascorbic acid synthesis is 2-keto-L-gluconic acid (2-KLG). Conventional industrial synthesis of 2-KLG involves 2 successive fermentations: i) Glucose → 2,5-diketo-D-gluconic acid (in *Erwinia herbicola*), followed by, ii) 2,5-diketo-D-gluconic acid → 2-KLG (in *Corynebacterium sp.*). A *Corynebacterium sp.* 2,5-diketo-D-gluconic acid reductase gene was cloned into *E. herbicola* resulting in a novel strain capable of fermentation of glucose into 2-KLG in a single stage [51].

### 6.2. Engineering metabolic shifts to increase synthesis of desired end-products

The introduction of genes encoding secondary metabolites within a host capable of producing its own different secondary metabolites can result in the construction of an array of enzymatic activities capable of producing novel products [51]. Antibiotics mederrhodins A and B, dihydrogranatirhodin, 2-nonrerythromycins A, B, C and D, and isovaleryl spiramycin are all produced within recombinant strains of *Streptomyces* engineered utilizing this strategy [51].

The manipulation of metabolic fluxes towards synthesis of a desired end-product is likely the strategy most amenable to biofuels production. The synthesis of desired products typically involves metabolic flow past forks where intermediates may enter alternative pathways [7, 40, 52, 53]. Maximizing the synthesis of valuable products therefore requires the desired pathways to be made a priority and alternative pathways be minimized to the extent possible without inhibiting cell viability.

Over-expression of an enzyme, or of enzymes, catalyzing a reaction towards the desired fork is a common strategy in metabolic engineering [11]. Heterologous gene expression of pyruvate decarboxylase and alcohol dehydrogenase from *Zymomonas mobilis* within *C. cellulolyticum* was shown to increase cell density by 180% and cellulose consumption by 150% compared to wild type strains [54]. Acetate and ethanol were shown to increase by 93% and 53% respectively and H_2_ yields increased by more than 75%. High carbon flux had previously been shown to cause pyruvate overflow as a result of the inability of POR to metabolize pyruvate to acetyl-CoA. This overflow was shown to lead to cell growth inhibition and a flux towards increased ethanol and lactate production [41]. The introduction of these genes was hypothesized to increase NAD+ cycling via the alcohol dehydrogenase, which in turn promoted flux towards ATP producing pathways and, as a consequence, increased cell growth [Guedon *et al*. [54]. These results demonstrate that fermentation of cellulose can be improved by using genetically engineered strains of clostridia designed to favour production of industrially valuable metabolites.

More frequently antisense RNA (asRNA) strategies are being utilized to direct metabolic flow [55]. In contrast to the conventional introduction of a heterologous gene resulting in the expression of a novel protein, the aim of antisense RNA is to specifically down-regulate expression of a native protein and thus redirect metabolism. Down-regulation is achieved through the 1) inhibition of translation due to the duplex RNA structure impeding access to a ribosome binding site, 2) rapid degradation of the mRNA due to RNA-duplex specific RNases, and 3) inhibition of mRNA transcription as a result of premature termination [55]. Antisense RNA strategies offer a number of advantages over traditional gene inactivation techniques. Most notably, asRNA avoids the hazard of introducing lethal mutations, as complete inhibition of protein expression is unlikely. Furthermore, asRNA strategies may be used to inducibly repress protein expression through the utilization of inducible promoters for asRNA transcription [56].

The application of asRNA technology as a strategy to influence primary metabolism was first demonstrated by van den Berg *et al*. [57] during which they examined the role of a periplasmic hydrogenase within *Desulfovibrio vulgaris* during lactate fermentation. More recently asRNA has been used to direct metabolic flow within *Clostridium acetobutylicum* by reducing levels of enzymes responsible for butyrate formation [55]. Butyrate is produced from the intermediate butyryl coenzyme-A in two steps: i) Butyryl coenzyme- A → Butyryl phosphate (via phosphotransbutyrylase (PTB), followed by ii) Butyryl phosphate + ADP → Butyrate + ATP (via butyrate kinase (BK).

The genes *ptb* and *buk*, encoding PTB and BK respectively, are organized in a single *ptb*-*buk* operon. Previous studies have shown butyrate to influence induction of solventogenesis and it was therefore hypothesized that the down-regulation of PTB and BK would impact primary metabolism [55]. It was found that recombinant strains exhibited 70 and 80 % reduced PTB and BK activities respectively compared to the wild type strain. Growth yields of the recombinant strains were 28% less than controls. While levels of acid production were not affected in the *ptb* targeted strain (pRD1), acetone and butanol yields were 96% and 75 % lower respectively compared with the control strain. Finally, both lactate yields and lactate dehydrogenase activity were higher (∼100 % and ∼ 300 % respectively) [55].

Metabolic engineering of *C. acetobutylicum* strains ATCC 824 (pRD4) and ATCC 824 (pRD1) demonstrated that asRNA can be used down-regulate specific protein production and alter the metabolism of *C. acetobutylicum*. These studies, however, also suggest that the elaborate nature of metabolic regulatory systems makes the down-regulation of a specific pathway unlikely to reduce synthesis of a specific product [55]. These findings further stress the importance that design decisions should be based on a detailed understanding of the genetic, enzymatic and thermodynamic mechanisms that direct carbon flow. While relatively simple strategies may be successful in the development of novel bacterial strains, the dynamic nature of metabolism makes accurate forecasts of change problematic.

## 7. Conclusions

Biofuels are an attractive alternative to current petroleum-based fuels as they can be utilized as transportation fuels with little change to current technologies and have significant potential to improve sustainability and reduce greenhouse gas emissions. The transition from a fossil fuel-based economy to a biofuel-based economy depends on the improvement of biofuel yields via the successful development of suitable microorgansims capable of efficiently fermenting a variety of sugars while simultaneously displaying tolerance to high end-product concentrations. Cellulose represents an attractive feedstock for biofuels production because of its abundance, cost and ability to be efficiently degraded by cellulolytic bacteria.

Metabolic engineering of bacterial and yeast species has already demonstrated that the construction of novel strains using recombinant DNA technologies can confer advantageous traits in regards to biofuels production. Success, however, will be dependent upon design decisions based on a detailed understanding of the extremely complex genetic, enzymatic, and thermodynamic mechanisms that direct carbon flow. In combination with other strategies including (meta)genomics, biodiversity studies and systems biology, metabolic engineering is a promising approach to the improvement of biofuel yields and the establishment of renewable, non-polluting energy sources.

## Figures and Tables

**Table 1.** Common ethanolic motor-fuel formulations

Fuel^a^	Ethanol content (% v/v)
Hydrous ethanol (Alcohol)(Brazil)	95.5
E85 (North America)	85
Gasoline (Brazil)	24
E10 (gasohol)(North America)	10
Oxygenated fuel (USA)	7.6
Reformulated gasoline (USA)	5.7
Biodiesel' (Sweden)	15

a Hydrous ethanol contains 4.5 % (v/v) water. The other formulations contain hydrocarbons and a trace of water
